# The Predictive Efficacy of Tumor Mutation Burden (TMB) on Nonsmall Cell Lung Cancer Treated by Immune Checkpoint Inhibitors: A Systematic Review and Meta-Analysis

**DOI:** 10.1155/2021/1780860

**Published:** 2021-03-13

**Authors:** Zhang Nan, Wang Guoqing, Yu Xiaoxu, Mi Yin, He Xin, Li Xue, Wang Rong

**Affiliations:** ^1^Department of Clinical Laboratory, First Teaching Hospital of Tianjin University of Traditional Chinese Medicine, China; ^2^Tianjin Key Laboratory of Oral and Maxillofacial Function Reconstruction; Tianjin Stomatological Hospital; Hospital of Stomatology, Nankai University, China; ^3^Tianjin Central Hospital of Gynecology Obstetrics, China; ^4^Department of Radiotherapy, First Affiliated Hospital of Zhengzhou University, China; ^5^School of Medical Laboratory, Tianjin Medical University, China

## Abstract

**Background:**

Nonsmall cell lung cancer (NSCLC) is the most common type of lung cancer, and the majority of NSCLC patients are diagnosed at the advanced stage. Chemotherapy is still the main treatment at present, and the overall prognosis is poor. In recent years, immunotherapy has developed rapidly. Immune checkpoint inhibitors (ICIs) as the representative have been extensively applied for treating various types of cancers. Tumor mutation burden (TMB) as a potential biomarker is used to screen appropriate patients for treatment of ICIs. To verify the predictive efficacy of TMB, a systematic review and meta-analysis were conducted to explore the association between TMB and ICIs.

**Method:**

PubMed, EMBASE, Cochrane Library, and son on were systematically searched from inception to April 2020. Objective response rate (ORR), progression-free survival (PFS), and overall survival (OS) were estimated.

**Results:**

A total of 11 studies consisting of 1525 nonsmall cell lung cancer (NSCLC) patients were included. Comparison of high and low TMB: pooled HRs for OS, 0.57 (95% CI 0.32 to 0.99; *P* = 0.046); PFS, 0.48 (95% CI 0.33 to 0.69; *P* < 0.001); ORR, 3.15 (95% CI 2.29 to 4.33; *P* < 0.001). Subgroup analysis values: pooled HRs for OS, 0.75 (95% CI 0.29 to 1.92, *P* = 0.548) for blood TMB (bTMB), 0.44 (95% CI 0.26 to 0.75, *P* = 0.003) for tissue TMB (tTMB); for PFS, 0.54 (95% CI 0.29 to 0.98, *P* = 0.044) and 0.43 (95% CI 0.26 to 0.71, *P* = 0.001), respectively.

**Conclusions:**

These findings imply that NSCLC patients with high TMB possess significant clinical benefits from ICIs compared to those with low TMB. As opposed to bTMB, tTMB was thought more appropriate for stratifying NSCLC patients for ICI treatment.

## 1. Introduction

Lung cancer is cancer-related mortality's leading cause among all other types of cancers in both males (24%) as well as females (23%) in 2019 world-wide [1]. All lung cancers, approximately 80–85%, were diagnosed as nonsmall cell lung cancer (NSCLC) type. The three traditional approaches of cancer therapy involved surgery, radiotherapy, and chemotherapy. With the noteworthy evolution of oncological therapies over the past few decades, several novel therapy advancements have been established, which included targeted therapy, interventional therapy, and immunotherapy [2].

As an innovative therapy, immunotherapy has turned into a hot spot in cancer treatment's field. The principal research direction is presently the immune checkpoint inhibitors (ICIs), which are specially represented by programmed death 1 (PD-1) and programmed death-ligand 1 (PD-L1) inhibitors. ICIs have brought in a spectacular revolutionization in the treatment of diverse cancer types [3], notably in melanoma and NSCLC [4, 5]. The response rates unfortunately even remain to be low (approximately 20%) in unselected patient populations in most of the cancer types, and the cost that is associated with these therapies stays high (closed to $150,000 per patient annually) [6]. Although PD-L1 that has been completely assessed by immunohistochemistry (IHC) in tumor cells has been approved by the U.S. Food and Drug Administration (FDA) as the only predictive biomarker for selecting patients that undergo ICI, evaluation of outcomes of treatment in NSCLC [7] revealed PD-L1 was not a perfect biomarker. Several advanced NSCLC patients have shown primary resistance and only NSCLC patients about 20–30% benefit from PD-1/PD-L1 inhibitors with long-term responses [8]. So, it is crucial and pressing to find sensitive and specific biomarkers for screening patients that are treated by ICIs out.

Previous studies have reported that tumor-specific neoantigens are connected with increased immunogenicity [9], therefore, speculating that the tumors that present a high number of neoantigens might respond better to immunotherapy [10–12]. Recently, tumor mutational burden (TMB) acts as an indirect measure of tumor-derived neoantigens and emerges as a potential biomarker for ICI patient stratification. TMB is defined as the total number of DNA mutations per megabases (Mb) [13]. The clinical utility of TMB in NSCLC patients that were treated by ICIs has been reported by very few seminal studies [14–16]. In all these studies, high TMB showed correlation with longer clinical benefits such as increasing objective response rate (ORR), longer progression-free survival (PFS), and overall survival (OS) by treatment with ICIs. Controversy, however, still existed. OAK cohorts and POPLAR confirmed that regardless of the selected cutoff values, TMB still failed to distinguish patients with OS benefits after immunotherapy [17, 18]. Inconsistent consequence was likewise observed from a recent retrospective study with a small sample size [19], suggesting that a high TMB status is instead associated with worse clinical outcomes after immunotherapy. The controversial results for TMB have appealed to widespread attention from clinicians, and whether TMB plays a reliable predictive biomarker of immunotherapy demands further exploration.

Hence, a comprehensive systematic review and meta-analysis were carried out to evaluate TMB's effect quantitatively as a predictive biomarker in NSCLC patients that were treated by ICIs.

## 2. Materials and Methods

### 2.1. Literature Search and Eligibility Criteria

This systematic review and meta-analysis were performed according to the Preferred Reporting Items for Systematic Reviews and Meta-Analyses (PRISMA) guidelines [20]. The PubMed, EMBASE, Cochrane Library, SinoMed, and CNKI were systematically searched to identify relevant studies published from inception to April 2020 without any limitation to language. Two investigators (Zhang and Wang) independently searched the databases. The key search terms were as follows: (“tumor mutation burden” OR “tumor mutation load” OR “TMB”) AND (“non-small cell lung cancer” OR “NSCLC”) AND (“immune checkpoint inhibitors” OR “ICIs” OR “immune checkpoint blockade” OR “ICB”). A manual search was also conducted to find applicable studies from the references and related citations.

The studies were considered eligible if they met the following criteria: (1) patients diagnosed with NSCLC and accepted ICIs treatment; (2) TMB is defined exactly. The TMB of the included patients was calculated and assessed by whole exome sequencing (WES) or a hybrid capture-based targeted next generation sequencing (NGS) panel, both of which are available in clinical practice; (3) studies reporting either PFS and/or ORR and/or OS that compared with low TMB versus high TMB or report PFS and/or ORR and/or OS that compared with ICIs treatment and chemotherapy (any kind of chemotherapy) in low TMB group or high TMB group; (4) studies with survival data calculated as hazard ratio (HR) and 95% confidence interval (CI); and (5) only recent studies or studies with more complete information were selected when authors from the same institution published multiple articles by overlapping of the same data.

Reviews, notes, letters, editorials, comments, meeting abstracts, case reports, and cell or animal studies were excluded due to insufficient information.

### 2.2. Data Extraction and Quality Assessment

Two investigators (Zhang and Wang) independently extracted the data from the studies included. For baseline characteristics, the information regarding the first author, publication year, number of participants, study design, region, age, treatment, the cutoff value of TMB, detection method of TMB, and the main reporting outcomes were collected. For pooled analysis, HR and 95% CI for OS and/or PFS were extracted. Any disagreements were conferred and resolved by discussing with another author (Yu).

To assess the overall quality of the included studies, the Newcastle–Ottawa Scale (NOS) [21], Cochrane collaboration's tools [22], and methodological index for nonrandomized studies (MINORS) [23] were applied for assessing the risk of bias for retrospective studies, randomized controlled trials, and single-arm trial, respectively. Each study was evaluated by two independent reviewers (Zhang and Wang). In the NOS, the studies were assessed by three scales including selection, comparability, and outcome. The total NOS scores ranged from 0 to 9, and a higher score is associated with higher quality. In the Cochrane collaboration's tools, seven items including random sequence generation, allocation concealment, blinding of participants and personnel, blinding of outcome assessment, incomplete outcome data, selective reporting, and other bias evaluated the studies, and each item was categorized as low, high, or unclear risk of bias. In the MINORS, studies were assessed through eight items such as a clearly stated aim, inclusion of consecutive patients, prospective collection of data, appropriate endpoints related to the aim of the study, unbiased assessment of the study endpoint, follow-up period appropriate to the aim of the study, loss to follow-up of less than 5%, and prospective calculation of the study size, and each item was scored as 0 (not reported), 1 (reported but inadequate), or 2 (reported and adequate).

### 2.3. Statistical Analysis

The following meta-analyses were performed: (1) comparison of clinical benefits between low TMB group and high TMB group, (2) subgroup analysis based on different sample sources for TMB detection, and (3) comparison of ICIs with chemotherapy both in low TMB and high TMB group. The pooled HRs for PFS and/or OS and relative risk (RR) for ORR were also calculated. The heterogeneity among different trials was evaluated by using Cochrane's *I*^2^ statistics. *I*^2^ > 50% and/or *P* ≤ 0.10 indicates significant heterogeneity, and then a random-effects model was applied; otherwise, a fixed-effects model was used [24]. Sensitivity analyses were performed to explore the source of heterogeneity. All statistical analyses were conducted using Stata software (version 14.0; Stata Corporation, College Station, TX), and *P* < 0.05 was considered to be statistically significant.

## 3. Results

### 3.1. Search Results and Studied Characteristics

**Figure 1:**
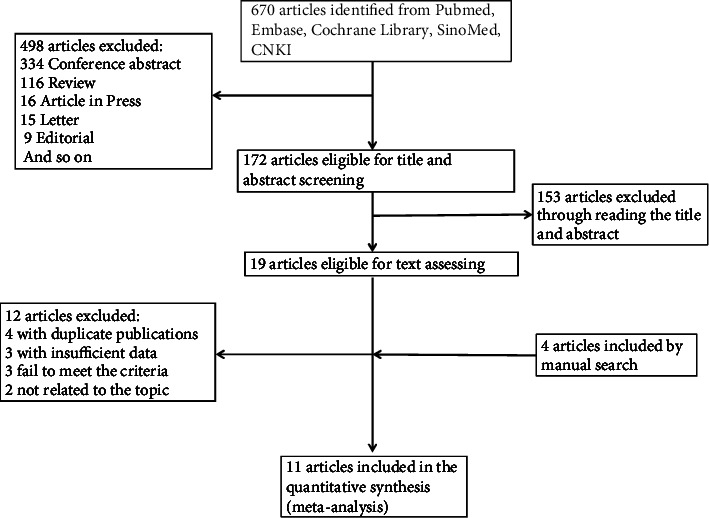
Flow chart of literature search.

A total of 670 publications were yielded by our initial search. After omitting reviews, letters, conference abstracts, and so forth, 172 publications were found eligible through screening the abstracts and titles. Of these, 153 publications were further ruled out. After 19 publications' full-text review and manual search of articles, 11 studies [14, 19, 25–33] were eventually included in the meta-analysis, and publication' years ranged from 2018 to 2020. The details regarding the studies' selection are outlined in the flow diagram of Figure 1. The principal characteristics of studies that are included in this meta-analysis are summed up in Table 1. Among the 11 included studies, 9 were retrospective studies, 1 was randomized controlled trial, and 1 was a single-arm trial, including 1525 NSCLC patients. The detection approach for TMB consisted of NGS and WES. Low and high TMB's definitions were heterogeneous among the studies. The cutoff value for defining high versus low TMB ranged from 9 to 243. Among all the included studies, Wang [27] and Wang (OAK and POPLAR) employed distinct research objects in the same study.

### 3.2. Quality Assessment

All the 11 studies were evaluated by Cochrane collaboration's tools, MINORS, and NOS. All the included studies proved moderate to high quality, and the outcomes were shown in Tables 2–4.

### 3.3. TMB Predictive Efficacy in Patients with High TMB versus Low TMB by ICI Treatment

**Figure 2:**
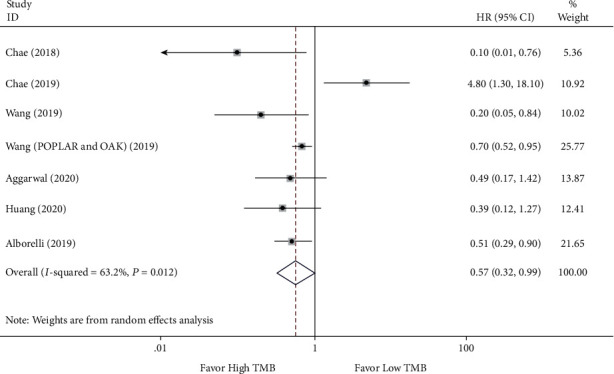
Forest plot of immune checkpoint inhibitors therapy for overall survival (OS) in high TMB group versus low TMB group.

**Figure 3:**
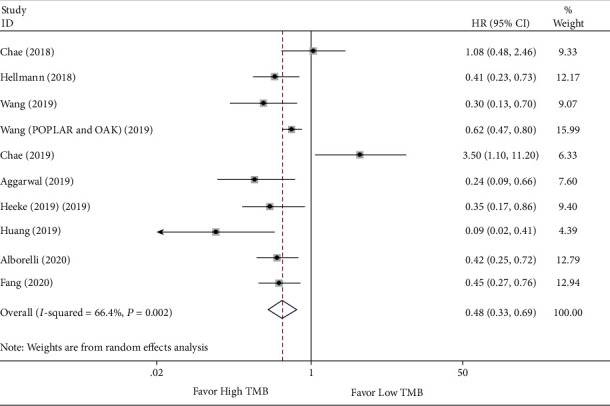
Forest plot of immune checkpoint inhibitors therapy for progression-free survival (PFS) in high TMB group versus low TMB group.

**Figure 4:**
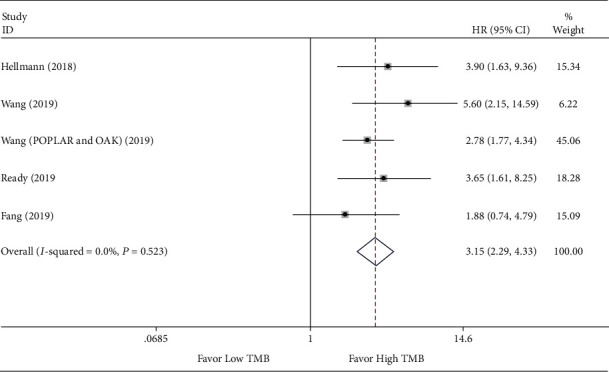
Forest plot of immune checkpoint inhibitors therapy for objective response rate (ORR) in high TMB group versus low TMB group.

To confirm TMB's efficacy as a predictor of ICI therapy, the clinical benefits of NSCLC patients with high TMB versus low TMB were evaluated. The HRs with 95% CI for PFS and OS were individually extracted. There are 6 studies [19, 25, 27, 28, 31, 32] with 7 sets of data for measuring pooled HR for OS and 9 studies [19, 25–28, 30–33] with 10 sets of data for measuring pooled HR for PFS. The meta-analysis results indicated that the pooled HR for OS was 0.57 (95% CI 0.32 to 0.99; *P* = 0.046), and the heterogeneity test was *I*^2^ = 63.2% (*P* = 0.012) (see Figure 2). Meantime, the pooled HR for PFS was 0.48 (95% CI 0.33 to 0.69; *P* <0.001), and the heterogeneity test was *I*^2^ = 66.4% (*P* = 0.002) (see Figure 3). Due to heterogeneity among the two analyses, random effects model was applied. The ORR between the low TMB group and the high TMB group was subsequently measured. As illustrated in Figure 4, there were 4 studies [26, 27, 29, 33] comprised 5 sets of data in this analysis. The merged RR for ORR was 3.15 (95% CI 2.29 to 4.33; *P* < 0.001), heterogeneity test *I*^2^ = 0.0% (*P* = 0.523), and so a fixed-effects model was applied. Overall, the above-pooled results pointed out that PFS, ORR, and the OS were significantly ameliorated in NSCLC patients with high TMB while compared to those with low TMB.

### 3.4. Influence of Sample Source on TMB Predictive Efficacy and Subgroup Analysis

In the light of the impact of sample sources (blood and tumor tissue) on TMB detection, a subgroup analysis was executed basis on bTMB and tTMB. The pooled HRs for OS were 0.75 (95% CI 0.29 to 1.92, *P* = 0.548) and 0.44 (95% CI 0.26 to 0.75, *P* = 0.003), respectively, for bTMB subgroup and tTMB. The heterogeneity test *I*^2^ was 74.3% (*P* = 0.009) in bTMB subgroup and 4.6% (*P* = 0.351) in tTMB subgroup. In contrast with OS, PFS was improved to a certain extent both in the bTMB and tTMB subgroup. The pooled HRs were 0.54 (95% CI 0.29 to 0.98, *P* = 0.044; bTMB subgroup) and 0.43 (95% CI 0.26 to 0.71, *P* = 0.001; tTMB subgroup), respectively. Heterogeneity tests, i.e., the *I*^2^ of the two subgroup analyses were 75.2% (*P* = 0.003, bTMB subgroup) and 56.6% (*P* = 0.056, tTMB subgroup) (Table 5). In summary, these results proposed that tTMB as a predictive biomarker could easily screen NSCLC patients that are appropriate for ICI therapy out compared to bTMB.

### 3.5. Efficacy Comparison for ICIs versus Chemotherapy and Subgroup Analysis according to the Level of TMB

**Figure 5:**
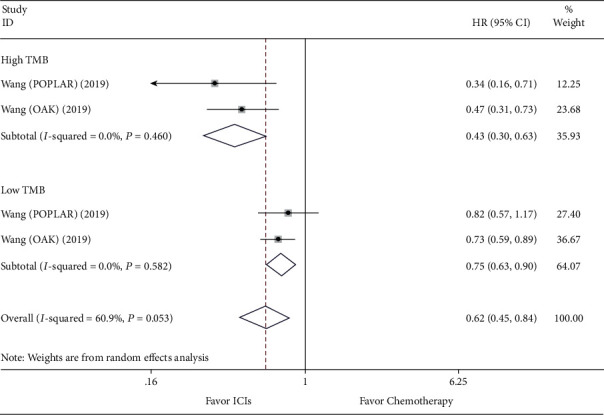
Forest plot of overall survival (OS) for comparing ICIs with chemotherapy and subgroup analysis.

**Figure 6:**
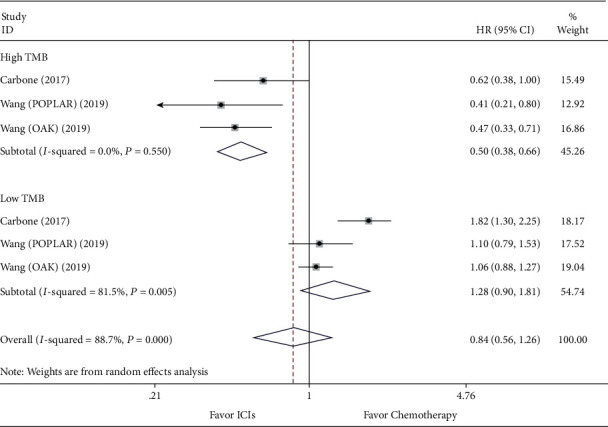
Forest plot of progression-free survival (PFS) for comparing ICIs with chemotherapy and subgroup analysis.

To assess ICI treatment's efficacy, ICIs were compared with chemotherapy. The pooled HRs for OS and PFS were 0.62 (95% CI 0.45 to 0.84, *P* = 0.002) and 0.84 (95% CI 0.56 to 1.26, *P* = 0.387), respectively. The heterogeneity tests shown *I*^2^ = 60.9% and 88.7%. Pursuant to TMB's distinct levels, a subgroup analysis was performed. The outcome of the subgroup analysis showed that the pooled HRs for OS was 0.43 (95% CI 0.3 to 0.63, *P* < 0.001) in high TMB group and 0.75 (95% CI 0.63 to 0.90, *P* = 0.002) in low TMB group. No meaningful heterogeneity was detected in the two groups (high TMB group: *I*^2^ = 0.0%, *P* = 0.46; low TMB group: *I*^2^ = 0.0%, *P* = 0.582), and so a fixed-effects model was applied (see Figure 5). For PFS, the pooled HRs were 1.28 (95% CI 0.90 to 1.81; *P* = 0.163; heterogeneity *I*^2^ = 81.5%, *P* = 0.002) and 0.50 (95% CI 0.38 to 0.66; *P* < 0.001; heterogeneity *I*^2^ = 0.0%, *P* = 0.550) in low TMB group and high TMB group. So, random-effects models and fixed-effects models were separately applied (see Figure 6). Above all, ICI therapy can ameliorate NSCLC patients' long-term survival status significantly as compared to chemotherapy. Particularly for patients with high TMB, the effect is more obvious.

### 3.6. Sensitivity Analysis

**Figure 7:**
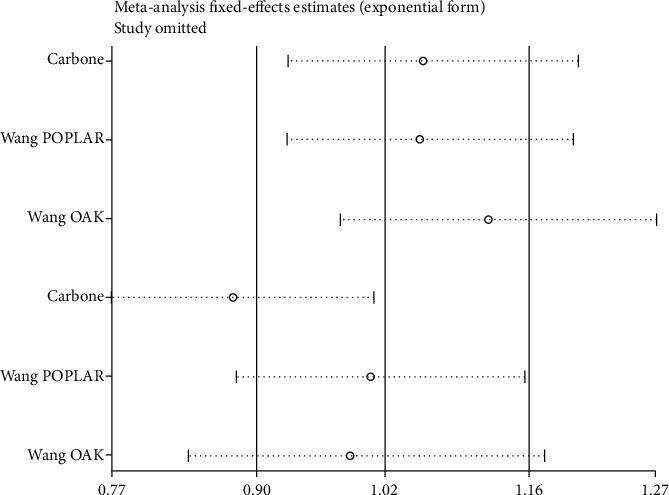
Sensitivity analysis of progression-free survival (PFS) for comparing ICIs with chemotherapy and subgroup analysis.

**Figure 8:**
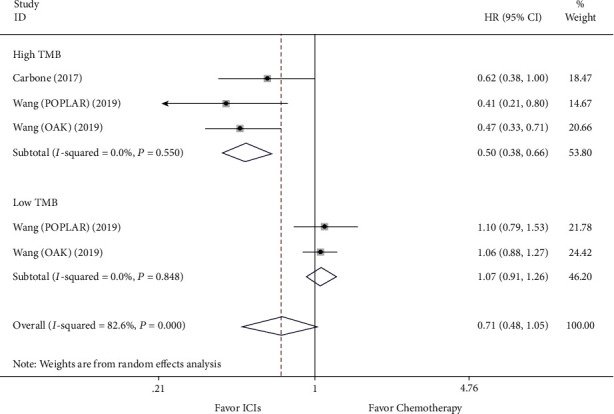
Forest plot of progression-free survival (PFS) for comparing ICIs with chemotherapy after moving out the heterogeneity source and subgroup analysis.

Sensitivity analysis was done to examine heterogeneity's potential source. Individual data impacting the outcome of ICIs' analysis compared to chemotherapy in the PFS was noticed (see Figure 7). When taking away the article [14], the result still continued to be unchanged, suggesting that the analysis was stable (see Figure 8).

## 4. Discussion

Immunotherapy has been widely applied in clinical practice for treating various types of cancers, especially NSCLC. Merely few patients, however, have benefitted from this promising treatment [34]. It, therefore, is pressing to identify a feasible and reliable predictive biomarker to stratify patients for ICI personalized treatment. In our study, a total of 11 trials that comprising 1525 NSCLC patients were collected for evaluating TMB as a predictive biomarker. The parameters for PFS, ORR, and OS were compared between low TMB patients and high TMB patients. The pooled results pointed out that the NSCLC patients with high TMB acquired longer-term survival benefits as compared to low TMB in PFS, ORR, and OS. TMB as an effective predictive biomarker to stratify patients for ICIs therapy possesses a certain feasibility. These results are consistent with that of Kim reported tumor mutational burden and efficacy of immune checkpoint inhibitors [35]. A potential interpretation for this might be that tumors with high TMB can carry higher neoantigen loads [36]. The neoantigens are novel protein epitopes that are specific to tumors that may be presented on the tumor cell surface by major histocompatibility complex molecules [37, 38]. A part of neoantigens is recognized as “non-self,” bringing about the T-cell activation and a tumor-targeted immune response [9, 39]. The immune checkpoint molecules in tumor cell surface, however, can regulate T-cell activation negatively, leading to neoantigen-driven immune responses' suppression and permitting tumor to escape immune surveillance [36]. Immune checkpoint inhibitors, such as anti-PD-1 and anti-PD-L1, can recover the antitumor immune response, leading to tumor cell eradication. So, tumors with high TMB are more probable to respond to immune checkpoint inhibitors [13, 40]. What is unforeseen is that the interval referral range for OS (95% CI: 0.32, 0.99) is so closed to 1; pulling this conclusion could be hazardous to a certain extent. Further study, especially for prospective clinical trail, to enlarge the sample size is consequently necessary.

Koeppel et al.'s study [41] reported that while ctDNA-WES was compared to tissue WES, the sensitivity only was 53%. Another study [42] likewise pointed out that the correlation was fairly disappointing when comparing bTMB with tTMB. The reason for this might be owning to concordance's lack between the two. In the meantime, Kim et al. [43] reported no significant OS benefits between bTMB-L subgroups and bTMB-H after immunotherapy. In view of these controversial consequences, a subgroup analyses for assessing the influence of tissue samples and blood on the outcomes was executed. The subgroup analysis results implied that tTMB appeared to be more reflective of long-term clinical benefits as compared to bTMB, particularly for OS, and was thought to be more appropriate as a predictor for screening patients through ICI treatment. BTMB, however, seemed not. Previous literature [44] is searched to discover one important reason for bTMB's inadequate predictive capability compared to tTMB as it demands a minimal amount of ctDNA. The tumors must shed DNA into the blood, if ctDNA could be detected in the blood for optimal assay performance. But studies [45, 46] have released that 20%-30% of the tumors had nonshedding samples, and Stage I and II tumors shed less DNA generally into the bloodstream than more advanced and metastatic tumors [45, 47]. So, the assay limit of detection from blood sample could cause an important source of discrepancy. Another important reason for the discordance between tTMB and bTMB is due to technical variations. Stetson [48] has reported low allele frequency (AF) (<1%) samples can cause false positive and false negative results easily. So, variants with less than 1% variant AF ought to be viewed with caution, particularly provided that they are novel variants that are not previously reported. But it is not deniable that a case [19] in our analysis presented an exceptionally large HR value and 95% CI and could produce certain influence to the final results. Although tumor tissue sample is regarded as the standard starting material to perform analysis, its disadvantage is likewise apparent, especially in NSCLC, due to unavailable or inadequate. BTMB assessment from ctDNA, however, is a very attractive approach with less invasive nature, more readily available source of material for examination, and less tumor heterogeneity biases. Although bTMB implementation in a routine setting is confronting all sorts of challenges, the news which EGFR and KRAS have been approved for clinical use by FDA based on plasma-based genotyping tests [49] bring some expectation. With the uninterrupted efforts of a consortium of academia, industry, regulatory agencies, and policymakers, it comes true eventually in routine clinical practice.

Besides, the comparison of ICIs with chemotherapy and subgroup analysis that was based on TMB's different levels was performed. These results confirmed that the NSCLC patients through ICI treatment are shown better OS than that through chemotherapy treatment. In particular, OS and PFS for patients with high TMB have shown a significant clinical improvement. Due to not adequate data, the ORR was not able to analyze. Meanwhile, there was likewise no meaningful difference for PFS in the low TMB subgroup. ICI treatment's efficacy, however, cannot be disregarded particularly in patients with high TMB. On the contrary, these results imply that NSCLC patients with high TMB treated by ICIs were more probable to obtain long-term clinical benefits in comparison with those with low TMB. Immunotherapy may be a better choice for NSCLC patients with high TMB. But we also acknowledge that more elaborate data on immunotherapy versus chemotherapy need to be supplemented. Due to available data's lack, we have not been capable to clarify whether immunotherapy is a first-line or second-line treatment compared to standard treatment. On 16 June 2020, based on the results of KEYNOTE-158 [50], FDA approved pembrolizumab for the treatment of adult and pediatric patients with unresectable or metastatic tumor mutational burden-high (TMB-H) [≥10 mutations/megabase (mut/Mb)] solid tumors, as determined by an FDA-approved test, that have progressed following prior treatment and who have no satisfactory alternative treatment options [51]. Although results of keynote-158 suggest that immunotherapy may be the final choice, there is not sufficient data to support this approach for NSCLC. On the contrary, several reports [52, 53] have demonstrated that immunotherapy as first-line therapy possesses more efficacy and less toxicity compared to chemotherapy. These consequences are consistent with our conclusion that immunotherapy does illustrate more advantages over chemotherapy. This news further supports our point of view that TMB as a predictive biomarker for screening appropriate patients out is feasible and reliable. It is not deniable that the predictive role likewise needs to be dealt with caution. After all, the number of studies that are included in this meta-analysis is comparatively less, and likewise, meta-analysis' power is not sufficient. So, these consequences ought to be validated in the next trials.

TMB as a predictor, however, is far from perfect. There are some limitations and other issues in TMB's use in routine clinical practice. Previous studies have disclosed several promising biomarkers for forecasting ICIs' efficacy, like PD-1/L1expression [54], neutrophil-lymphocyte ratio (NLR) [55], tumor infiltrative lymphocytes [56], and mismatch repair deficiency (MRD) [57]. Each biomarker possesses its own weakness and strength. Maybe, multiple biomarkers' combination might be a more excellent alternative in predicting the ICIs' efficacy or risk of acquiring resistance in NSCLC patients.

There, however, are some limitations in our meta-analysis. First of all, the heterogeneity throughout the studies is significant. Our consequences were based on unadjusted analysis, and more accurate outcomes would result from adjustments for eliminating other interference factors such as gender, age, PD-L1 status, treatment, detection method, and TMB cut-off value. Although subgroup analysis was played to decrease heterogeneity, the results unfortunately demonstrated no significant improvement. Second, less number of trials were involved in this analysis, and the sample size varied among the included studies, which could bring out potential publication bias. Third, the studies that are included in the analysis are generally retrospective in nature, which may introduce selection bias inherently and other uncontrolled variables could impact the assessment of associated clinical outcomes and TMB. Finally, pursuant to the Cochrane Collaboration Handbook [58], testing for publication bias is not recommended if the number of involved studies is fewer than ten, and so funnel plot and Egger linear regression test were not conducted.

## 5. Conclusions

NSCLC patients with high TMB possessed significant clinical benefits from ICIs as compared to those with low TMB. As opposed to bTMB, tTMB is a more excellent choice for screening appropriate NSCLC patients through ICI treatment. Despite practical and technical barriers exist, TMB might even be a feasible and reliable predictive biomarker.

## Figures and Tables

**Table 1:** The main characteristics of studies included in the meta-analysis.

Study	Year	Type of study	*N*	Region	Age (year)	Experiment drugs	Line of therapy	Detection method	TMB cutoff value	Sample source	Outcome
Carbone et al. [14]	2017	Randomized	530	Multiple areas	63 (32-89)	Nivolumab	1st	WES	243	Tissue	PFS
Chae et al. [25]	2018	Retrospective	35	USA	64.5 (37-88)	ICIs	NA	NGS	15	Tissue	PFS, OS
Hellmann et al. [26]	2018	Retrospective	75	USA	66 (42-87)	Nivolumab plus ipillimumab	1st	WES	158	Tissue	PFS, ORR
Wang et al. [27]	2019	Retrospective	64	China	58 (8)	ICIs	All	NGS	12	Blood	PFS, OS
Wang et al. (POPLAR and OAK) [27]	2019	Retrospective	429	Multiple areas	NA	Atezolizumab	2st or 3st	NGS	12	Blood	PFS, OS, ORR
Chae et al. [19]	2019	Retrospective	27	USA	NA	ICIs	All	NGS	14.5	Blood	PFS, OS
Aggarwal et al. [28]	2019	Prospective	52	USA	NA	Pembrolizumab	1st	NGS	16	Blood	PFS, OS
Ready et al. [29]	2019	Single arm	98	USA, Canada	65.5 (42-85)	Nivoluman plus ipillimumab	1st	NGS	10	Tissue	ORR
Heeke et al. [30]	2019	Retrospective	48	France	NA	Pembrolizumab or nivolumab	1st or 2st	NGS	10	Tissue	PFS
Huang et al. [31]	2019	Retrospective	16	China	61 (34-86)	ICIs	All	NGS	10	Tissue	PFS, OS
Alborelli et al. [32]	2020	Retrospective	76	Switzerland	66 (31-90)	ICIs	All	NGS	9	Tissue	PFS, OS
Fang et al. [33]	2020	Retrospective	75	China	54 (28-73)	ICIs	NA	NGS	10	Tissue	PFS, ORR

**Table 2:** The Newcastle-Ottawa Scale (NOS) assessment for risk of bias of the included studies.

Study	Selection	Comparability	Outcome	Total score^∗^
Chae et al. [25]	3	1	2	6
Hellmann et al. [26]	3	1	3	7
Wang et al. [27]	4	1	3	8
Wang et al. (POPLAR and OAK) [27]	3	1	2	6
Chae et al. [19]	3	1	3	7
Aggarwal et al. [28]	3	1	3	7
Heeke et al. [30]	3	1	2	6
Huang et al. [31]	4	1	3	8
Alborelli et al. [32]	4	1	3	8
Fang et al. [33]	3	1	3	7

^∗^NOS points: 0 to 3: very high risk of bias; 4 to 6: high risk of bias; 7 to 9: low risk of bias.

**Table 3:** The Cochrane collaboration's tool for assessing risk of bias of the included studies.

Study	Random sequence generation	Allocation concealment	Blinding of participants and personnel	Blinding of outcome assessment	Incomplete outcome data	Selective reporting	Other bias
Carbone et al. [14]	Low risk	Low risk	High risk	Low risk	Low risk	Low risk	Low risk

**Table 4:** Methodological index for nonrandomized studies for risk of bias.

Items	Score^∗^
(1) A clearly stated aim	2
(2) Inclusion of consecutive patients	2
(3) Prospective collection of data	2
(4) Endpoints appropriate to the aim of the study	2
(5) Unbiased assessment of the study endpoint	0
(6) Follow-up period appropriate to the aim of the study	2
(7) Loss to follow up less than 5%	1
(8) Prospective calculation of the study size	2

^∗^This method assessed the risk of bias of Ready's study. The items are scored 0 (not reported), 1 (reported but inadequate), or 2 (reported and adequate).

**Table 5:** Results of subgroup analysis of blood sample group versus tissue sample group.

Subgroup	Overall survival					Progression-free survival				
	Number of study estimates	HR (95% CI)	*P* value	*I* ^2^ (%)	*I* ^2^ among subgroups (%)	Number of study estimates	HR (95% CI)	*P* value	*I* ^2^ (%)	*I* ^2^ among subgroups (%)
All studies	6	0.57 (0.32, 0.99)	0.046	63.2%		9	0.48 (0.33, 0.69)	<0.001	66.4%	
Blood sample	3	0.75 (0.29, 1.92)	0.548		74.3%	4	0.54 (0.29, 0.98)	0.044		75.2%
Tissue sample	3	0.44 (0.26, 0.75)	0.003		63.2%	5	0.43 (0.26, 0.71)	0.001		56.6%

## Data Availability

The datasets that are used and analysed during the current study are available from the corresponding author on reasonable request.
